# Development of a sensitive Luminex xMAP-based microsphere immunoassay for specific detection of *Iris yellow spot viru*s

**DOI:** 10.1186/s12985-018-0952-4

**Published:** 2018-04-04

**Authors:** Cui Yu, Cuiyun Yang, Shaoyi Song, Zixiang Yu, Xueping Zhou, Jianxiang Wu

**Affiliations:** 1 Shanghai Entry-Exit Inspection and Quarantine Bureau, Shanghai, 200135 China; 2 Institute of Biotechnology, Zhejiang University, Hangzhou, 310058 China; 3 Institute of Plant Protection, Chinese Academy of Agriculture Sciences, Beijing, 10094 China

**Keywords:** *Iris yellow spot virus*, Antibody, Microsphere immunoassay, Detection

## Abstract

**Background:**

*Iris yellow spot virus* (IYSV) is an *Orthotospovirus* that infects most *Allium* species. Very few approaches for specific detection of IYSV from infected plants are available to date. We report the development of a high-sensitive Luminex xMAP-based microsphere immunoassay (MIA) for specific detection of IYSV*.*

**Results:**

The nucleocapsid (*N*) gene of IYSV was cloned and expressed in *Escherichia coli* to produce the His-tagged recombinant N protein. A panel of monoclonal antibodies (MAbs) against IYSV was generated by immunizing the mice with recombinant N protein. Five specific MAbs (16D9, 11C6, 7F4, 12C10, and 14H12) were identified and used for developing the Luminex xMAP-based MIA systems along with a polyclonal antibody against IYSV. Comparative analyses of their sensitivity and specificity in detecting IYSV from infected tobacco leaves identified 7F4 as the best-performed MAb in MIA. We then optimized the working conditions of Luminex xMAP-based MIA in specific detection of IYSV from infected tobacco leaves by using appropriate blocking buffer and proper concentration of biotin-labeled antibodies as well as the suitable ratio between the antibodies and the streptavidin R-phycoerythrin (SA-RPE). Under the optimized conditions the Luminex xMAP-based MIA was able to specifically detect IYSV with much higher sensitivity than conventional enzyme-linked immunosorbent assay (ELISA). Importantly, the Luminex xMAP-based MIA is time-saving and the whole procedure could be completed within 2.5 h.

**Conclusions:**

We generated five specific MAbs against IYSV and developed the Luminex xMAP-based MIA method for specific detection of IYSV in plants. This assay provides a sensitive, high-specific, easy to perform and likely cost-effective approach for IYSV detection from infected plants, implicating potential broad usefulness of MIA in plant virus diagnosis.

## Background

Orthotospoviruses infect a wide range of plant species and are among the most serious threats to vegetable crops. *Iris yellow spot virus* (IYSV) is an *Orthotospovirus* in the family Tospoviridae of the order Bunyavirales, which has been reported to infect most *Allium* species such as onion crops and some ornamentals such as Spiny Sowthistle, Irises and Lisianthus [1–3]. IYSV was firstly recorded in onion crops in Idaho, USA in 1989 [4] and then in the Netherlands in 1992, where it was characterized to be a new, distinct orthotospovirus from *Iris hollandica* [5]. Subsequently, IYSV was found to occur in [6] Brazil [7], Australia [8], Japan [9], Chile [10], Spain [11], Guatemala [12], Peru [13], India [14], and Egypt [15].

The symptoms caused by IYSV in *Allium* spp. include yellow- to straw-coloured, diamond-shaped lesions on infected leaves and flowering scapes [1]. Diamond-shaped lesions are particularly pronounced on infected scapes and gradually merge along the disease progresses, eventually leading to the lodging of infected scapes. In seed crops, this would lead to a severe reduction in yield and quality. Early to mid-season infection in bulb crops results in reduced vigour and bulb size [16]. To date, two thrips species, *Frankliniella fusca* and *Thrips tabaci* (Thysanoptera: Thripidae), have been experimentally proved to be able to transmit IYSV [17]. Nowadays, IYSV has been emerging as a severe threat to the onion crop in the world [1, 16].

One of the prerequisites to develop the management strategies for controlling Orthotospoviruses is to develop accurate diagnose and identification methods. Unlike *Tomato spotted wilt virus* (TSWV), IYSV infections typically do not become systemic in onion or other host species. The enzyme-linked immunosorbent assay (ELISA)-based technique has been developed for IYSV detection [18]; however, ELISA assays are time-consuming, laborious and error-prone due to its low sensitivity and potential false negative results. Nucleic acid-based approaches such as RT-PCR and real-time PCR are also available for detecting and identifying IYSV [13, 19], but the use of PCR-based approaches for the high-throughput detection of IYSV is uneasy and time-consuming in practice when a large number of samples are handled simultaneously.

In the past several years, the microsphere immunoassay (MIA) based on the xMAP (Flexible multi-analyte profiling) technology has emerged as an alternative for detection of microbial pathogens. This system involves covalent coupling of an antigen or antibody on carboxy-lated polystyrene microspheres that are internally dyed with a fluorophore, which consists of as many as 100 unique beads/microspheres and has the potential for simultaneous (multiplex) detection of 100 different targets [20]. The detection of microspheres is implemented by two lasers, one for identifying the color of the microsphere and determining the type of the objects, another for determining the intensity of the microspheres and the quantity of the objects. To date, several studies that use this technology to detect multiple targets in plants have been reported [21–23]. However, the xMAP-based MIA method for detection of *Orthotospoviruses* (including IYSV) has not been available yet.

In this study, we report the generation of a set of monoclonal antibodies (MAbs) and polyclonal antibody against IYSV. Based on these antibodies, we developed a Luminex xMAP-based MIA for high-throughput detection of IYSV. We compared the sensitivity and specificity of MIA with the conventional triple antibodies-based sandwich (TAS)-ELISA in detecting IYSV, and demonstrated that the MIA method we developed show better performance.

## Methods

### Virus isolates

The virus sources of IYSV, TSWV and *Impatiens necrotic spot virus* (INSV) were kindly supplied by Professor R. Kormelink (Wageningen Agricultural University, The Netherlands). The viruses were maintained and multiplicated in *Nicotiana benthamiana* plants by our laboratory as previously described. Positive controls of IYSV, TSWV and INSV were purchased from DSMZ (Germany). *Arabis mosaic virus* (ArMV), *Tobacco ringspot virus* (TRSV) and *Strawberry latent ringspot virus* (SLRSV) were used for validating the specificity of the microsphere immunoarray.

### Cloning and expression of the *N* gene of IYSV in *Escherichia coli*

To clone the *N* gene of IYSV, a pair of primers FCP-F (CCATATGGATGTCTACCGTTAGGGTGAAACC, the *Nde* I site was underlined) and FCP–R (CAAGCTTGTTAATTATATCTATCCTTCTTGGAGG, the *Hind* III site was underlined) were designed and synthesized based on the reported genomic sequence of IYSV (*N* gene, GenBank accession No. AB505813). Total RNAs were extracted from IYSV-infected tobacco plants using RNeasy Plant mini Kit (Qiagen, Hilden, Germany), and RT-PCR was performed using the One-Step RT-PCR system (Takara, Dalian, China) according to the manufacturer’s instruction using the primers FCP-F and FCP-R. PCR products were purified using gel extraction columns (Qiagen, Hilden, Germany). The purified PCR product was digested with *Nde* I and *Hind* III, and cloned into 6 × His-tagged prokaryotic expression vector pCold II (Novagen, Darmstadt, Germany). The recombinant expression vector pCold II-IYSV-N was transformed into *Escherichia coli* pG-TF2 strain (GE Healthcare, Bucks, UK). And recombinant N protein was produced by induction with 1 mM isopropylthio-b-galactosidebovine (IPTG) at 15 °C overnight. The recombinant N protein was purified using Ni-NTA agarose as instructed by the manufacturer (Qiagen, Hilden, Germany).

### Production of polyclonal antibody

Two rabbits were immunized with purified IYSV recombinant N protein. For the first subcutaneous injection, 0.8 mg purified recombinant N protein in 1 ml phosphate-buffered saline (PBS) was emulsified with an equal volume of complete Freund’s adjuvant (Sigma-Aldrich). Subsequently, the recombinant protein (0.5 mg in 1 ml PBS) emulsified with an equal volume of incomplete Freund’s adjuvant (Sigma-Aldrich) was administered at 3-week interval for three times. Rabbits were bled 1 week after the fourth injection, and the sera were used in TAS-ELISA.

### Preparation of hybridomas secreting MAbs

Intraperitoneal immunizations of 25 μg purified recombinant protein were administered to four femal BALB/c mice with complete Freund’s adjuvant for the primary immunization and incomplete Fruend’s adjuvant for subsequent boosts. Hybridomas secreting MAbs against IYSV recombinant nucleocapsid protein were produced by fusion of spleen cells from immunized BALB / c female mice and the mouse myeloma cell line SP2/0 as previously described [24]. Hybridoma supernatants were screened for the presence of specific antibodies against recombinant N protein as detected by an indirect-ELISA as described by Jiang et al. [25]. The specific positive hybridoma cells were cloned with the method of the limiting dilution [26] for more than three times to get the hybridoma lines that produce the MAb against the N protein of IYSV.

### Production of MAbs in ascitic fluids

Hybridoma cells were injected intraperitoneally into mineral oil-primed BALB/c mice to produce the ascites. At 7–10 days after the injection the ascitic fluids were collected and tested for their titre by indirect-ELISA [27] using purified recombinant N protein of IYSV as antigen. The isotypes of the MAbs were determined by double immunodiffusion assay with the mouse MAb isotyping reagents according to the manufacturer’s instruction (Sigma-Aldrich). The specificity of the MAbs was further determined by Western blot analysis using IYSV-infected and healthy plant tissues as the antigens and negative controls, respectively.

### SDS-PAGE and Western blot

The purified recombinant N protein was analyzed by sodium dodecyl sulfate-polyacrylamide gel electrophoresis (SDS-PAGE) followed by either.

Coomassie blue R-250 staining or Western blot analysis after transferred to a nitrocellulose membrane (GE Healthcare) using a Trans-blot SD Semi-Dry transfer Cell (Bio-Rad). IYSV MAbs or anti-His tag MAb (1:1000, Novagen) was used for Western blot analysis.

### ELISA

Detection of viruses was carried out following the standard procedures TAS-ELISA [28]. The sample was considered to be positive when its OD_405_ value was greater or equal to 2 times of the mean value from negative controls. Each sample was independently repeated two times.

### Coating of magnetic beads with polyclonal antibody and coupling biotin to MAbs

The purified antibodies from the rabbit polyclonal IYSV were coupled with specific MagPlex microsphere Set (BioRad) according to the instruction manual. Briefly, about 2000 microsphere beads were washed with activation buffer twice and then activated with Sulfo-N-hydrosuccinimide (sulfo-NHS) and N- [3-dimethylaminopropyl]-N′- ethylcarbodiimide hydrochloride (EDC) by shaking for 20 min at room temperature. Approximately 12 μg antibodies were added to the activated beads and incubated 2 h at room temperature. The success of the coupling reaction was evaluated by detection of the coupled rabbit IgG with a phycoerythrin-conjugated goat anti-rabbit antibody. The MAbs against IYSV were biotinylated with the Thermo Fisher EZ-Link Sulfo-NHS-LC-Biotin Reagent (Cat. No. PI-21335) according to the standard procedure provided by the manufacture.

### Microsphere immunoassay

The whole process of MIA detection was carried out in a 96-well plate (Tellgen, Shanghai, China). The samples were prepared by grinding 0.1 g fresh tissue or 0.01 g lyophilized tissue in 1 mL PBS with BSA (PBS-TBN, 8 g NaCl/L, 0.2 g/L KCl, 1.44 g/L Na_2_HPO4 and 0.24 g/L KH_2_PO_4_, with BSA 1%, pH 7.4). The samples were centrifuged at 14,000 g for 5 min and the supernatant was placed in a new tube. The microspheres coated with the antibody were vortexed for 30 s at full speed and then diluted in PBS -TBN to prepare the work solution at 8 × 10 ^4^ beads/mL. In order to be detected with the X-map analyzer, the biotinylated MAbs were also coupled with streptavidin R-phycoerythrin (SA-RPE) in the assay. To do this, the concentration of the MAbs labeled with biotin was adjusted to about 20 μg/ mL and labeled with SA-RPE with the ratio 1: 0.2 at 4 °C for 1 h.

To establish the assay for detection of IYSV, 25 μL PBS-TBN, 10 μl sample saps and 25 μL SA-RPE-labeled antibodies were mixed and reacted at 37 °C for 5 min. Then, 25 μL beads work solution was added and the reactions were continued for 60 min at 37 °C. The median fluorescent intensity (MFI) of each reaction was measured and analyzed with 100 beads per well, using the Bio-plex 200 system (Bio-Rad).

### Comparison between ELISA and MIA for detecting IYSV

The sensitivity of TAS-ELISA and MIA in detecting IYSV was evaluated with a same set of samples. Crude extracts from IYSV-infected leaf tissues were diluted with crude extracts from non-infected tobacco leaves to generate a dilution series. A same set of samples were then tested by TAS-ELISA and MIA, respectively. Buffer and negative controls were included in each set of reactions to verify the reaction performance.

## Results

### Production of recombinant IYSV N proteins in *E. coli*

**Fig. 1:**
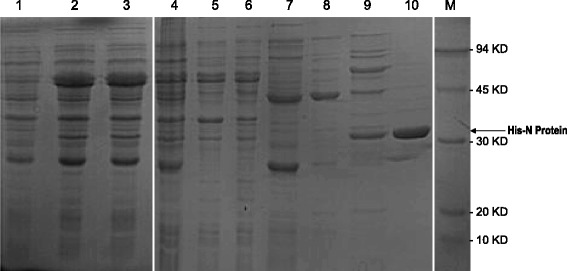
SDS-PAGE analysis of recombinant N protein of IYSV. Lane 1: Non-induced crude; Lane 2: pColdII-IYSV in pG-TF2 clone1 induced crude; Lane 3: pColdII-IYSV in pG-TF2 clone2 induced crude; Lane 4: Load sample; Lane 5:Flowthrough; Lane 6:Flowthrough-2; Lane 7:Elution by 50 mM Imidazole; Lane 8: Elution by 50 mM Imidazole; Lane 9: Elution by 200 mM Imidazole; Lane 10:Elution by 500 mM Imidazole

The 822-bp full-length *N* gene of IYSV was amplified by RT-PCR using the total RNAs extracted from the IYSV-infected tobacco leaves. The sequence confirmed N gene was then subcloned into the protokarytic expression vector pCold II and introduced into the *E.coli* strain pG-TF2. After induction by adding IPTG, SDS-PAGE and Western blot analysis confirmed that the recombinant N protein was properly expressed in the *E.coli* strain pG-TF2 harboring pCold II-IYSV-N (Fig. 1). The His-tagged recombinant N proteins were affinity-purified using Ni-NTA agarose and eluted by imidazole.

### Generation of MAbs against recombinant IYSV N proteins

To generate the highly specific MAbs against IYSV, we used 25 μg recombinant N proteins to immunize the mice. After 6 months, five hybridoma cell lines (7F4, 11C6, 12C10, 14H12, 16D9) secreting MAbs against recombinant IYSV N proteins were obtained. Immunodiffusion assay showed that four MAbs (7F4, 12C10, 14H12, 16D9) belonged to the subclass IgG1, whereas 11C6 was classified into the subclass IgG 2a. The light chains of all the five MAbs belonged to k-chain.

We then determined the titres of ascitic fluids of MAbs by TAS- ELISA and found they were ranged from 10^− 5^ to 10^− 7^. The IgG yields of these five MAbs from ascitic fluids were also determined, with the MAb 14H12 showing the highest yield of 42.5 mg/ml and the 16D9 producing the least (20.33 mg/ml) (Table 1).

**Table 1:** Properties of MAbs against recombinant N protein of IYSV

MAbs	Isotype	Titre	Yield of IgG in ascitic fluid(mg/mL)
7F4	IgG1, κ chain	10^− 5^	35.25
11C6	IgG2a,κ chain	10^−6^	20.57
12C10	IgG1, κ chain	10^−7^	26.33
14H12	IgG1, κ chain	10^− 7^	42.50
16D9	IgG1, κ chain	10^−7^	20.33

**Fig. 2:**
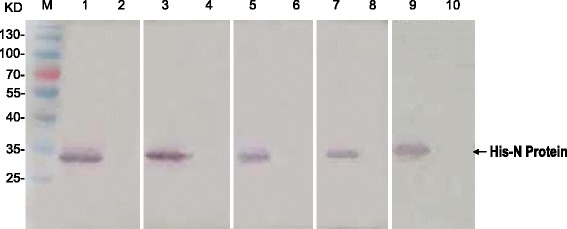
The five MAbs specifically recognize the recombinant N protein of IYSV in Western blot. M, protein molecular weight marker; Lanes 1,3,5,7, and 9 were loaded with the protein extracts from the *E. coli* strain expressing the recombinant N protein, but the lanes 2,4,6,8, and10 were loaded with protein extracts from the *E. coli* strain expressing the empty vector. 1,2 were detected by 16D9; 3,4 were detected by 11C6; 5,6 were detected by 7F4; 7,l8 were detected by 12C10; 9,10 were detected by 14H12

To test the specificity of the MAbs we obtained, we carried out the Western blot analyses. As shown in Fig. 2, all the five MAbs could strongly and specifically detect the recombinant IYSV N proteins from total extracts of *E. coli*, producing the sole band with molecular weight of approximately 32 kDa, indicating that the five MAbs we obtained could specifically recognize the recombinant IYSV N protein.

### Development and optimization of MIA in detecting IYSV

Previous study has demonstrated that some antibodies can be used for ELISA assays but may be not suitable for development of the MIA [29]. Therefore, the identification of an appropriate antibody against the target is critical for developing a reliable and practical MIA assay. To determine which MAbs against IYSV is more suitable for the MIA detection, we coupled microspere with the IYSV rabbit antibodies and labeled MAb with biotin, respectively. The results showed that the rabbit antibody-coated microsphere and SA-RPE-coupled 7F4 had high MFI value to sample and low MFI value to buffer control, while the MAbs 11C6 and 12C10 followed in the performance. Thus, we subsequently used the MAb 7F4 to optimize the MIA assay. We adjusted the concentration of 7F4-biotin and the quality ratio between the 7F4-biotin and streptavidin R-phycoerythrin (SA-RPE), and established that the optimal concentration of biotin-labeled 7F4 was 6 μg/mL and the quality ratio between the biotin-labeled 7F4 and SA-RPE is 1:0.3. Then, we evaluated the specificity of MIA in detecting IYSV by conducting comparative assays. Three *Nepoviruses* including ArMV, TRSV and SLRSV as well as two other Orthotospoviruses including INSV and TSWV were used. We found that the MIA system could only detect the leaf samples infected by IYSV, but not those infected by other six viruses (Table 2), indicating that the MIA method we developed has high specificity in detecting IYSV.

**Table 2:** The specificity of MIA in detecting IYSV

Virus										
	ArMV	TRSV	SLRSV	INSV (R.K)	INSV (DSMZ)	IYSV (DSMZ)	IYSV (R.K)	TSWV (R.K)	TSWV (DSMZ)	Control
MFI value	55	99	64	47	71	2916	25,110	86	54	62

### The sensitivity of MIA in detecting IYSV

Both ELISA and MIA are based on the reaction between the antibody and antigen. To compare the sensitivity of ELISA and MIA in detecting IYSV, a same set of 2-fold serial dilutions of IYSV-infected plant saps and the MAb 7F4 were used for detection by ELISA and MIA, respectively. The results showed that the ELISA assay could detect the IYSV from the 1:40 dilution of plant saps. In contrast, MIA could detect the IYSV from the 1:640 dilution of plant saps as the lowest, indicating that the sensitivity of MIA in detecting IYSV is 16-fold higher than that of ELISA (Table 3).

**Table 3:** The sensitivity of ELISA and MIA in detecting IYSV

samples	Dilution	MFI value	
		MIA	ELISA
Inoculated plant	1:10	20,813	0.577
Inoculated plant	1:20	19,897	0.374
Inoculated plant	1:40	17,767	0.24
Inoculated plant	1:80	9865	0.178
Inoculated plant	1:160	5214	0.126
Inoculated plant	1:320	1212.5	0.110
Inoculated plant	1:640	233.5	0.122
Inoculated plant	1:1280	183	0.09
Healthy control	/	98.0	0.082

## Discussion

Although MIA has been widely used for detecting animal viruses and other pathogens, its development and application for detecting plant viruses is very limited to date. With regard to the methods for detecting IYSV, ELISA and PCR-based assays for detection of IYSV have been reported [18, 19, 30], but the highly sensitive and specific approaches for large-scale detection of IYSV are not reported in the literature, likely because of the lack of the specific antibodies against IYSV. In this study, we obtained the polyclonal antibody and five specific MAbs against IYSV (11C6, 12C10, 14H12, 7F4, and 16D9) by using the recombinant N protein of IYSV produced in *E. coli* (Fig. 1 and Table 1). The amino acid sequences of N protein from different IYSV isolates have high similarity, and thus the IYSV N protein is relatively conserved [12, 31, 32]. With these antibodies, we developed the TAS-ELISA and MIA methods for detecting IYSV. Compared with the TAS-ELISA, we found that the sensitivity of MIA is much higher (16-fold) in detecting IYSV from the extracts of infected tobacco leaves when the same antibodies were used in both assays (Table 3). Among the five MAbs we obtained, the MAb 7F4 showed the best performance in MIA detection of IYSV in terms of the specificity and efficiency (Table 2). Thus, to obtain the antibodies against the target viruses or pathogens, particularly the specific MAbs with high sensitivity, is the prerequisite in establishing the MIA for detection of plant viruses.

In addition to the increased detection sensitivity, compared with TAS-ELISA, the MIA method we developed offers several other advantages. First, the MIA procedure is less time-consuming, because the whole process of MIA in detecting IYSV from infected plant leaves could be completed in about 2.5 h. However, the normal TAS-ELISA process would require 6–8 h for obtaining the final results. Second, the MIA method we developed for detecting IYSV showed high specificity. Typically, the specificity of MIA is predominantly determined by the specificity of the antibodies used and the procedure used for enriching the viruses or pathogens from the infected tissues [33]. To develop a high-specific MIA method for detecting IYSV, we used the polyclonal antibody to coat the magnetic beads for enrichment of IYSV from the extracts of infected leaves. We also generated the MAbs against IYSV and obtain theMAb 7F4 that shows high sensitivity and specificity to ISYV by performing the comparative analysis. In addition, the optimized conditions were set up to couple biotin and SA-RPE with the MAb 7F4. It is likely that the enrichment procedure, high specificity of 7F4 and optimized conditions together make the MIA method we developed with the high specificity. Indeed, the MIA we developed could specifically detect IYSV from the extracts of infected tobacco leaves, but not three Nepoviruses including ArMV, TRSV and SLRSV and two other Orthotospoviruses including INSV and TSWV (Table 2), given that most isolates of IYSV have high nucleotide sequence identity [2, 3]. Thirdly, MIA can be automated for high-throughput detection using pipetting robots and the Luminex analyzer. The MIA procedure we developed is easy to perform and even without the pipetting robots a hundred of samples can be easily distributed into wells of the plate using the standard pipette within couple minutes. Fourth, undoubtedly, the Luminex xMAP-based MIA method possesses potential broad usefulness in plant virus detection. The MIA procedure we developed can be easily adapted for detecting the other plant viruses, provided that there are specific antibodies against the target viruses available. Compared with the TAS-ELISA, in principle the Luminex xMAP-based MIA method could also be used for simultaneous detection of multiple pathogens including mix-infected viruses, although this needs to be demonstrated by future investigations.

## Conclusions

We developed a Luminex xMAP-based MIA method for detection of IYSV in plants. We showed this is a sensitive, high-specific, easy to perform and cost-effective method. IYSV is a Orthotospovirus that infects most Allium species. Although both IYSV and TSWV, which is well-known as the most prevalent Orthotospovirus in the world, are transmitted by thrips, the occurrence of IYSV in the world is relatively scared and restricted in the Allium-growing countries/regions of the world. Its occurrence was also reported only in a few plant species. Thus, the MIA method we reported here would be particularly useful for detecting IYSV in plants, especially from the imported onions, to prevent its wide spread across the world.

## Abbreviations

ArMV: *Arabis mosaic virus*
ELISA: enzyme-linked immunosorbent assay
INSV: *Impatiens necrotic spot virus*
IPTG: isopropylthio-b-galactosidebovine
IYSV: *Iris yellow spot virus*
MAbs: monoclonal antibodies
MFI: median fluorescent intensity
MIA: microsphere immunoassay
N: nucleocapsid
PBS: phosphate-buffered saline
SA-RPE: streptavidin R-phycoerythrin
SLRSV: *Strawberry latent ringspot virus*
TAS: triple antibodies-based sandwich
TRSV: *Tobacco ringspot virus*
TSWV: *Tomato spotted wilt virus*
